# Work-family conflict and self-rated health trajectories among ELSA-Brasil workers: the moderating role of education

**DOI:** 10.47626/1679-4435-2024-1270

**Published:** 2025-01-07

**Authors:** Camila Arantes Ferreira Brecht D’Oliveira, Daniela Paula, Aline Silva-Costa, Susanna Toivanen, Luana Giatti, Odaleia Barbosa de Aguiar, Maria de Jesus Mendes da Fonseca, Rosane Harter Griep

**Affiliations:** 1 Health and Environmental Surveillance Secretariat, Ministry of Health, Brasília, DF, Brazil; 2 National School of Statistical Sciences, Instituto Brasileiro de Geografia e Estatística, Rio de Janeiro, RJ, Brazil; 3 Department of Mathematics and Statistics, Universidade do Estado do Rio de Janeiro (UERJ), Rio de Janeiro, RJ, Brazil; 4 Department of Epidemiology and Biostatistics, Universidade Federal Fluminense, Niterói, RJ, Brazil; 5 School of Health, Care and Social Welfare, Mälardalen University, Västerås, Sweden; 6 Brazilian Hospital Services Company, Hospital das Clínicas, School of Medicine, Universidade Federal de Minas Gerais, Belo Horizonte, MG, Brazil; 7 Instituto de Nutrição, UERJ, Rio de Janeiro, RJ, Brazil; 8 Department of Epidemiology and Quantitative Methods in Health, Escola Nacional de Saúde Pública, Fundação Oswaldo Cruz (FIOCRUZ), Rio de Janeiro, RJ, Brazil; 9 Environmental and Health Education Laboratory, Instituto Oswaldo Cruz, FIOCRUZ, Rio de Janeiro, RJ, Brazil

**Keywords:** work-life balance, social determinants of health, health status, gender and health, equilíbrio trabalho-vida, determinantes sociais de saúde, nível de saúde, saúde de gênero

## Abstract

**Introduction:**

Studies on the association between work-family conflict and self-reported health are mostly cross-sectional; few studies have investigated the effect of education on this association.

**Objectives:**

To investigate association between work-family conflict, family-work conflict, lack of time for self-care and leisure due to family and work demands, and self-rated health trajectories, examining sex differences and the modifying effect of education on these associations.

**Methods:**

Data from active workers (women = 4,283; men = 3,851) from the three waves and annual follow-up (2008-2020) of the Longitudinal Study of Adult Health were analyzed using multinomial logistic models.

**Results:**

Work-family conflict, family-work conflict, and lack of time were associated with worse self-rated health trajectories in both sexes. However, among women who reported a lack of time for self-care and leisure, education was a modifying factor. The odds of a fair or poor self-reported health trajectory were higher among women with a high education level who reported a lack of time “sometimes” or “often” than in women with a low education level.

**Conclusions:**

Work-family conflict dimensions were associated with worse self-reported health trajectories among both women and men. Education only modified this effect among women.

## Abstract (pt)

**Introdução:**

A maioria dos estudos sobre a associação entre o conflito trabalho-família e a autoavaliação de saúde é transversal, e poucos investigaram o efeito da escolaridade nessa associação.

**Objetivos:**

Investigar a associação entre o conflito do trabalho para família e da família para trabalho, a falta de tempo para autocuidado e lazer por demandas familiares e de trabalho e as trajetórias de autoavaliação de saúde, examinando diferenças de gênero e a modificação de efeito da escolaridade nessas associações.

**Métodos:**

Os dados dos trabalhadores ativos (mulheres = 4.283; homens = 3.851) das três ondas e o acompanhamento anual (2008-2020) do Estudo Longitudinal de Saúde do Adulto foram tratados por modelos logísticos multinominais.

**Resultados:**

O conflito trabalho-família e família-trabalho e a falta de tempo foram associados a piores padrões de trajetórias de autoavaliação de saúde em ambos os sexos. No entanto, apenas entre mulheres que relataram falta de tempo para o autocuidado e lazer a escolaridade se mostrou modificadora de efeito. Em comparação às mulheres com escolaridade baixa, aquelas com alto nível de escolaridade que relataram falta de tempo “às vezes” ou “frequentemente” tiveram maiores chances de pertencer às trajetórias de autoavaliação de saúde regular ou ruim.

**Conclusões:**

As dimensões do conflito entre trabalho e família foram associadas a piores trajetórias de autoavaliação de saúde entre mulheres e homens. A escolaridade modificou o efeito nas associações encontradas apenas entre as mulheres.

## INTRODUCTION

Work and family are the central domains of adult life, in which women and men carry out most of their daily activities and play roles conditioned by society.^1,2^ In this context, the concept of work-family conflict (WFC) is defined as a “a form of interrole conflict in which the role pressures from the work and family domains are mutually incompatible in some respect.”^1^ This type of conflict occurs when the ability to fulfill work demands is hindered by family demands or vice versa.^1,2^ Several studies have shown that WFC is related to physical and mental health.^3-5^

The relationship between WFC and self-rated health (SRH), which is widely used and considered an important predictor of morbidity and mortality,^6^ has been mainly evaluated in cross-sectional studies, showing associations between WFC and worse SRH.^6-11^ A study of Japanese workers found a stronger association between WFC and poor SRH among women than men.^10^ A review of European studies found conflicting results regarding sex differences and associations between WFC and several health outcomes, including SRH.^4^ A Brazilian study found an association between WFC and worse SRH among highly educated women,^12^ which suggests that education may modify this relationship. Thus, the results of cross-sectional studies indicate that sex and education are relevant factors in the associations between WFC and SRH.^4,11,12^

Longitudinal studies on SRH trajectories are still scarce. As far as we could find, only one international study has investigated the association between WFC and SRH trajectories.^13^ This study of 2,327 Swiss workers found that SRH trajectories tend to decline slowly over time, and this decline is associated with work burnout. Furthermore, workers with lower education levels who reported work burnout were more likely to have poor SRH trajectories than those with higher education levels.^13^

One mechanism that can explain the effect of WFC on health over time is stress, which, in the short term, can activate adaptation-related hormones. In the long term, this process can lead to physical and mental changes that contribute to the emergence of disease.^14^ Furthermore, WFC can favor unhealthy health behaviors, such as smoking, excessive alcohol consumption, and unhealthy eating, which can contribute to worsening SRH over time.^15,16^ Furthermore, a relationship between WFC and health-related behaviors, such as physical activity, diet, and smoking, was only observed among women.^17^

Thus, considering the scarcity of longitudinal studies on the association between WFC and SRH trajectories and the importance of sex and education in this relationship, the present study investigated the association between WFC, family-work conflict (FWC), lack of time for self-care and leisure due to family and work demands, and SRH trajectories in men and women. The modifying role of education in this association was also evaluated.

## METHODS

### STUDY DESIGN AND PARTICIPANTS

This study used data from the Longitudinal Study of Adult Health (*Estudo Longitudinal de Saúde do Adulto* - ELSA-Brasil), a prospective cohort study of civil servants from five public universities and one research institution in six Brazilian state capitals. The main objective of ELSA-Brasil was to identify risk factors associated with chronic health conditions.

The study included data from active participants in the three waves of ELSA-Brasil: baseline (2008-2010), wave 2 (2012-2014), and wave 3 (2017-2019), in addition to annual telephone monitoring interviews (2009 to Dec 28, 2020). The baseline wave had 15,105 participants, of whom 6,470 were retired and were excluded from the analyses (wave 1: n = 3,009; wave 2: n = 2,062; wave 3: n = 1,399) due to the lack of occupational information at baseline and the fact that the health behavior of those who retired during follow-up differs from active workers. Among the remaining 8,635 participants, the following categories were excluded due to limited numbers: self-declared Asians (n = 198), self-declared Indigenous (n = 74), those who died (n = 69), and those missing information on the variables of interest (n = 160). Thus, the final study population included 8,134 active workers (4,283 women and 3,851 men) who participated in the three waves of ELSA-Brasil. This study was approved by the Research Ethics Committees of all involved institutions, and all participants provided written informed consent.

### DATA COLLECTION

Data were collected through examinations and face-to-face interviews at the research centers, as well as through annual telephone follow-up. All stages were conducted by trained and certified teams according to standardized procedures.

### STUDY VARIABLES

#### Outcome: self-rated health trajectories

To measure SRH over time, participants answered the following question at each point of contact: “In general, compared to people your age, how do you consider your health status?”, with response options ranging from “very good” (1 point), “good” (2 points), “fair” (3 points), “poor” (4 points) to “very poor” (5 points).

SRH trajectories were defined through a latent class growth curve model, which uses longitudinal measurements to identify distinct latent classes that represent the heterogeneity of SRH trajectories intrinsic to the study population.^18^ Details about the construction of these patterns have been described elsewhere.^19^ SRH was measured at 11 time points for each participant (three waves and eight annual monitoring calls) between 2008 and 2020. The monitoring protocol identified three patterns of stable SRH trajectories over time. For each group, the average SRH was evaluated at each time point, ranging from 1 (very good) to 5 (very poor). According to the evolution of the mean, the trajectories were classified as “good”, “fair”, or “poor”.

#### Exposure: work-family conflict

WFC was measured at baseline using the Brazilian version of an international questionnaire,^1^ whose psychometric properties had already been demonstrated.^20^ The items assessed the extent to which work affects family time - “Work demands prevent you from spending the desired amount of time with your family” - and work stress - “Work demands make it difficult to fulfill household responsibilities, such as taking care of the house and children”. The questionnaire also assessed the extent to which family affects work - “Family demands interfere with professional responsibilities, such as arriving on time, completing tasks, not missing appointments, traveling for work, and attending meetings outside regular hours”. A fourth item measured the interference of work and family in personal care and leisure through the following statement: “Family and professional demands prevent you from spending the desired amount of time for your own care and leisure”. Responses were given on a five-point scale (“never to almost never”, “rarely”, “sometimes”, “often” and “very often”) and were grouped into three levels: “never” (combining “never to almost never” and “rarely” as the reference category); “sometimes”; and “often” (combining “often” and “very often”).^12^

### COVARIATES

Covariates were measured at baseline. The sociodemographic variables included: age (complete years), self-reported race (Black, mixed, or White), per capita net family income categorized in multiples of the minimum wage (≤ 3x, > 3x), based on 2008 levels (BRL 415.00; BRL 1.00 = USD 1.76), marital status (married/common-law relationship, separated/widowed, single); and education (≤ completed high school, university degree, or graduate degree).

Psychosocial work stress was measured using the Brazilian version of the Swedish Demand-Control-Social Support Questionnaire.^21^ This instrument assesses the interaction between psychological demand and control based on the median of the two dimensions divided into four quadrants: low work stress; active work; passive work; and high work stress. Social support was categorized as high or low, using the median as the cut-off point. The psychometric properties of the scale were deemed appropriate in the Brazilian context in a prior study.^21^

The presence of comorbidities was categorized as “yes” for participants who reported having a myocardial infarction, stroke, heart failure, hypertension, and/or diabetes or “no” for those reporting none of the above.

### STATISTICAL ANALYSIS

A descriptive analysis was performed, using means and standard deviations (SD) or absolute (n) and relative (%) values for the variables of interest. Multinomial logistic regression was then used to estimate the magnitude of the association between the four WFC dimensions and SRH trajectories; the “good” trajectory was considered the reference category. The socioeconomic and work variables were considered model adjustments. Comorbidities, which were considered mediating variables and closely related to the outcome, were not included in the models.

According to Akaike’s information criterion, removing the variables “marital status”, “work stress” and “social support” resulted in a better model fit. Therefore, the final model was adjusted for socioeconomic variables (age, income, race, and education). All analyses were stratified by sex and, to assess the effect of education, the multiplicative interaction of education (≤ high school; ≥ university) with the four WFC dimensions was tested; the only significant interaction was between education and lack of time for self-care among women. All analyses were performed in R 4.0.5, using the libraries “lcmm”, “nnet”, “tidyverse” and “factoextra”.

## RESULTS

Just over half of the participants were women (52.5%). The mean age of the women was 47.7 (SD, 6.5) years and that of men was 47.8 (SD, 6.7) years. The most prevalent sociodemographic characteristics among both men and women were White race, a high school education level, being married/having a common-law relationship, having a passive job, and never perceiving WFC. Women reported time-based and work stress-based WFC more frequently than men, in addition to lack of time for self-care and leisure (Table 1).

**Table 1:** Distribution of study variables among men and women according to self-rated health (SRH) trajectories, Longitudinal Study of Adult Health (ELSA-Brazil), 2008-2020

Variables	SRH trajectory							
	Total n = 4,283	Women			Total n = 3,851	Men		
		Good n = 1,260 (29.4%)	Fair n = 2,544 (59.4%)	Poor n = 479 (11.2%)		Good n = 1,110 (29.0%)	Fair n = 2,405 (62.0%)	Poor n = 336 (9.0%)
Mean age (SD)	47.7 (6.5)	47.7 (6.5)	47.9 (6.5)	47.9 (6.6)	47.8 (6.7)	47.2 (6.9)	47.9 (6.6)	48.9 (6.8)^*^
	n (%)	n (%)	n (%)	n (%)		n (%)	n (%)	n (%)
Race								
Black	795 (18.6)	182 (22.9)	482 (60.6)	131 (16.5)^*^	541 (10.0)	139 (25.7)	346 (63.9)	56 (10.4)^*^
Mixed	1,192 (27.8)	312 (26.2)	720 (60.4)	160 (13.4)	1,255 (32.6)	324 (25.8)	800 (63.7)	131 (10.5)
White	2,296 (53.6)	766 (33.4)	1,342 (58.4)	188 (8.2)	2,055 (53.4)	647 (31.5)	1,259 (61.3)	149 (7.2)
Education								
High school	1,781 (41.6)	368 (20.7)	1,138 (63.9)	275 (15.4)^*^	1,816 (41.1)	410 (22.6)	1,191 (65.6)	215 (11.8)^*^
University	894 (20.9)	259 (28.9)	556 (62.2)	79 (8.8)	542 (14.1)	145 (26.8)	370 (68.3)	27 (4.9)
Graduate	1,608 (37.5)	633 (39.4)	850 (52.9)	125 (7.8)	1,493 (38.8)	555 (37.2)	844 (56.5)	94 (6.3)
Per capita income‡								
≤ BRL 1,245.00	2,084 (48.6)	460 (22.1)	1,315 (63.1)	309 (14.8)^*^	1,929 (50.1)	439 (22.8)	1,274 (66.0)	216 (11.2)^*^
> BRL 1,245.00	2,199 (51.4)	800 (36.4)	1,229 (55.9)	170 (7.7)	1,922 (49.9)	671 (34.9)	1,131 (58.8)	120 (6.2)
Marital status								
With partner	2,437 (56.9)	724 (29.7)	1,460 (59.9)	253 (10.4)§	3,141 (81.6)	879 (27.9)	1,993 (63.5)	269 (8.6)§
Divorced/separated	1,257 (29.3)	352 (28.0)	735 (58.5)	170 (13.5)	483 (12.5)	151 (31.3)	289 (59.8)	43 (8.9)
Single	589 (13.7)	184 (31.2)	349 (59.3)	56 (9.5)	227 (5.9)	80 (35.2)	123 (54.2)	24 (10.6)
Stress at work								
Low stress	842 (19.7)	325 (38.6)	460 (54.6)	57 (6.8)^*^	966 (25.1)	333 (34.5)	576 (59.6)	57 (5.9)^*^
Active work	79 (17.7)	289 (38.1)	401 (52.8)	69 (9.1)	666 (17.3)	241 (36.2)	373 (56.0)	52 (7.8)
Passive work	1,634 (38.1)	407 (24.9)	1,025 (62.7)	202 (12.4)	1,561 (40.5)	395 (25.3)	1,014 (65.0)	152 (9.7)
High stress	1,048 (24.5)	239 (22.8)	658 (62.8)	151 (14.4)	658 (17.1)	141 (21.4)	442 (67.2)	75 (11.4)
Social support at work								
High	1,756 (41.0)	545 (31.0)	1,036 (59.0)	175 (10.0)§	1,822 (47.3)	559 (30.7)	1,119 (61.4)	144 (7.9)^§^
Low	2,527 (59.0)	715 (28.3)	1,508 (59.7)	304 (12.0)	2,029 (52.7)	551 (27.2)	1,286 (63.4)	192 (9.5)
Time-based WFC								
Often	1,623 (37.9)	459 (28.3)	989 (60.9)	175 (10.8)§	1,034 (26.8)	312 (30.2)	630 (60.9)	92 (8.9)
Sometimes	1,243 (29.0)	357 (28.7)	760 (61.1)	126 (10.1)	1,257 (32.7)	349 (27.8)	798 (63.5)	110 (8.7)
Never/rarely	1,417 (33.1)	444 (31.3)	795 (56.1)	178 (12.6)	1,560 (40.5)	449 (28.8)	977 (62.6)	134 (8.6)
Work stress-based WFC								
Often	1,090 (25.5)	289 (26.5)	644 (59.1)	157 (14.4)^*^	626 (16.3)	179 (28.6)	393 (62.8)	54 (8.6)
Sometimes	1,324 (30.9)	417 (31.5)	773 (58.4)	134 (10.1)	1,160 (30.1)	324 (27.9)	725 (62.5)	111 (9.6)
Never/rarely	1,869 (43.6)	554 (29.6)	1,127 (60.3)	188 (10.1)	2,065 (53.6)	607 (29.4)	1,287 (62.3)	171 (8.3)
FWC								
Often	272 (6.3)	64 (23.5)	162 (59.6)	46 (16.9)^*^	276 (7.2)	62 (22.5)	180 (65.2)	34 (12.3)§
Sometimes	1,118 (26.1)	302 (27.0)	679 (60.7)	137 (12.3)	1,035 (26.8)	290 (28.0)	646 (62.4)	99 (9.6)
Never/rarely	2,893 (67.6)	894 (30.9)	1,703 (58.9)	296 (10.2)	2,540 (66.0)	758 (29.8)	1,579 (62.2)	203 (8.0)
Lack of time								
Often	1,524 (35.6)	416 (27.3)	901 (59.1)	207 (13.6)^*^	976 (25.3)	277 (28.4)	600 (61.5)	99 (10.1)
Sometimes	1,432 (33.4)	424 (29.6)	871 (60.8)	137 (9.6)	1,259 (32.7)	353 (28.0)	794 (63.1)	112 (8.9)
Never/rarely	1,327 (31.0)	420 (31.7)	772 (58.2)	135 (10.2)	1,616 (42.0)	480 (29.7)	1,011 (62.6)	125 (7.7)
Comorbidities								
No	3,114 (72.7)	1,031 (33.1)	1,793 (57.6)	290 (9.4)^*^	2,366 (61.4)	817 (34.5)	1,415 (59.8)	134 (5.7)^*^
Yes	1,167 (27.3)	229 (19.6)	751 (64.4)	187 (16.0)	1,485 (38.6)	293 (19.7)	990 (66.7)	202 (13.6)

FWC = family-work conflict; WFC = work-family conflict; SD = standard deviation.

* p < 0.001.

‡ BRL = 1.00. USD = 1.76.

§ p < 0.05.

For both sexes, a poor SRH trajectory was more prevalent among Black or mixed-race participants and those with a lower education level, lower income, passive work, high stress at work, low social support, and comorbidities. A poor SRH trajectory was also more prevalent among women who reported frequent time-based or work stress-based WFC, frequent FWC, and frequent lack of time for self-care and leisure. Among men, only FWC was associated with a worse SRH trajectory. That is, a higher percentage of men who reported frequent conflict were classified in the fair or poor trajectories (Table 1).

Table 2 shows the results of the regression models adjusted for women. Women who reported frequent time-based conflict had a 37% higher chance (95% confidence interval [95%CI] 1.06-1.78) of having a poor SRH trajectory than those who reported never/rarely having this type of conflict. The odds of a fair (odds ratio [OR] = 1.29; 95%CI 1.10-1.54) or poor (OR = 2.11; 95%CI 1.62-2.76) SRH trajectory were higher among women with frequent work stress-based conflict than those who reported never/rarely having this type of conflict. Similar results were observed for women who reported frequent FWC (OR = 2.16; 95%CI 1.45-3.23 and OR = 2.19; 95%CI 1.45-3.30 for fair and poor SRH trajectories, respectively).

**Table 2:** Crude and adjusted associations between work-family conflict (WFC) dimensions and self-rated health (SRH) trajectories among women, Longitudinal Study of Adult Health (ELSA-Brazil), 2008-2020

WFC dimensions	SRH trajectory			
	Fair		Poor	
	OR_1_ (95%CI)	OR_2_ (95%CI)	OR_1_ (95%CI)	OR_2_ (95%CI)
Time-based WFC				
Sometimes	0.98 (0.83-1.16)	1.11 (0.94-1.33)	0.92 (0.70-1.20)	1.12 (0.85-1.47)
Often	0.83 (0.70-0.97)	1.00 (0.85-1.19)	1.05 (0.82-1.34)	1.37 (1.06-1.78)§
Work stress-based WFC				
Sometimes	0.91 (0.77-1.06)	1.04 (0.88-1.22)	0.94 (0.73-1.22)	1.17 (0.90-1.53)
Often	1.09 (0.92-1.30)	1.29 (1.10-1.54)§	1.60 (1.24-2.06)§	2.11 (1.62-2.76)§
FWC				
Sometimes	1.18 (1.00-1.38)	1.36 (1.07-1.74)§	1.36 (1.07-1.74)§	1.47 (1.15-1.88)§
Often	1.32 (0.98-1.79)	2.16 (1.45-3.23)§	2.16 (1.45-3.23)§	2.19 (1.45-3.30)§
Lack of time				
Sometimes	1.11 (0.94-1.31)	1.01 (0.76-1.32)	1.00 (0.76-1.32)	1.25 (0.94-1.65)
Often	1.17 (0.99-1.39)	1.55 (1.19-1.99)§	1.54 (1.19-1.99)§	2.20 (1.68-2.89)§

Good SRH trajectory = reference category.

FWC = family-work conflict; OR_1_ = odds ratio for crude associations; OR_2_ = odds ratio for associations adjusted for age, self-reported race, education, and income; 95%CI = 95% confidence interval.

§ p < 0.05.

Men who reported that they “sometimes” had work stress-based conflict had a 1.44 times higher odds (95%CI 1.08-1.91) of a poor SRH trajectory than those who reported having it “never/rarely”. The odds of a fair (OR = 1.42; 95%CI 1.05-1.93) or poor (OR = 2.04; 95%CI 1.29-3.22) SRH trajectory were higher among men who reported frequent FWC. The odds of a poor or fair SRH trajectory were higher among men who reported “sometimes” or “often” lacking time for self-care and leisure (Table 3).

**Table 3:** Crude and adjusted associations between dimensions of work-family conflict (WFC) and self-rated health (SRH) trajectories among men, Longitudinal Study of Adult Health (ELSA-Brazil), 2008-2020

WFC dimensions	SRH			
	Fair		Poor	
	OR_1_ (95%CI)	OR_2_ (95%CI)	OR_1_ (95%CI)	OR_2_ (95%CI)
Time-based WFC				
Sometimes	1.06 (0.88-1.24)	1.13 (0.95-1.35)	1.05 (0.79-1.40)	1.16 (0.86-1.55)
Often	0.92 (0.77-1.10)	1.10 (0.91-1.32)	0.98 (0.73-1.33)	1.25 (0.91-1.72)
Work stress-based WFC				
Sometimes	1.05 (0.89-1.24)	1.18 (0.99-1.39)	1.21 (0.92-1.60)	1.44 (1.08-1.91)§
Often	1.03 (0.84-1.26)	1.22 (0.99-1.51)	1.07 (0.75-1.51)	1.39 (0.97-2.00)
FWC				
Sometimes	1.05 (0.90-1.25)	1.16 (0.98-1.37)	1.27 (0.96-1.68)	1.50 (1.13-1.99)§
Often	1.39 (1.03-1.88)§	1.42 (1.05-1.93)§	2.05 (1.31-3.20)§	2.04 (1.29-3.22)§
Lack of time				
Sometimes	1.06 (0.90-1.26)	1.21 (1.02-1.43)§	1.21 (0.91-1.62)	1.54 (1.14-2.08)§
Often	1.02 (0.86-1.23)	1.29 (1.07-1.56)§	1.37 (1.01-1.85)§	2.14 (1.55-2.96)§

Good SRH trajectory = reference category.

FWC = family-work conflict; OR_1_ = odds ratio for crude associations; OR_2_ = odds ratio for associations adjusted for age, self-reported race, education, and income; 95%CI = 95% confidence interval.

§ p < 0.05.

A multiplicative interaction between lack of time for self-care and leisure and education level was found only among women (p < 0.05). Women with a high education level who reported “often” having a lack of time had a 1.56 times higher odds (95%CI 1.26-1.94) of a fair SRH trajectory and 3.88 times higher odds (95%CI 2.45-6.14) of a poor SRH trajectory than those who reported “never/rarely” lacking time. Furthermore, women with a high education level who reported “sometimes” having a lack of time for self-care and leisure had a 1.47 times higher odds (95%CI 1.18-1.83) of a fair SRH trajectory and a 2.12 times higher odds (95%CI 1.29-3.47) of a poor SRH trajectory than those who reported “never/rarely” lacking time (Table 4).

**Table 4:** Crude and adjusted associations between lack of time for self-care and leisure due to family and work demands and self-rated health (SRH) trajectories among women, Longitudinal Study of Adult Health (ELSA-Brazil), 2008-2020

Lack of time	SRH trajectory							
	Fair				Poor			
	Low education		High education		Low education		High education	
	OR_1_ (95%CI)	OR_2_ (95%CI)	OR_1_ (95%CI)	OR_2_ (95%CI)	OR_1_ (95%CI)	OR_2_ (95%CI)	OR_1_ (95%CI)	OR_2_ (95%CI)
Sometimes	1.00 (0.76-1.32)	0.99 (0.75-1.30)	1.44 (1.15-1.79)§	1.47 (1.18-1.83)§	0.91 (0.62-1.33)	0.89 (0.61-1.31)	2.00 (1.22-3.26)§	2.12 (1.29-3.47)§
Often	1.18 (0.87-1.58)	1.18 (0.87-1.59)	1.53 (1.24-1.90)§	1.56 (1.26-1.94)§	1.42 (0.97-2.00)	1.48 (1.01-2.20)§	3.65 (2.32-5.75)§	3.88 (2.45-6.14)§

Good SRH trajectory = reference category.

OR_1_ = odds ratio for crude associations; OR_2_ = odds ratio for associations adjusted for age, self-reported race, education, and income; 95%CI = 95% confidence interval.

§ p < 0.05.

## DISCUSSION

This study was an innovative investigation of the association between four WFC dimensions and different SRH trajectories, based on approximately 10 years of follow-up of a large sample of active public servants from institutions in six Brazilian state capitals. We also examined the effect of education on these associations.

The results showed that WFCs, whether time-based or work stress-based, were more relevant for women, with significant associations and a dose-response gradient for fair and poor SRH trajectories. FWC was associated with worse SRH trajectories in both sexes. Regarding the association between lack of time and SRH trajectory, education was an effect-modifying variable only for women. The odds of a worse SRH trajectory were higher among those with a high education level. Among men, a lack of time was associated with a worse SRH trajectory, regardless of education level.

Cross-sectional studies have shown an association between WFC and poorer SRH, which was more evident among women.^11,12^ FWC was also associated with poor SRH patterns among Japanese men and women, with the associations being more consistent among women.^10^ Thus, the sex differences we observed, i.e., more consistent associations between conflict and worse SRH trajectories for women, reinforce sex-related social roles, such as a greater responsibility for housework and child rearing among women. The literature indicates that women more often find it challenging to reconcile the demands of work and family due to these responsibilities.^22-24^

Despite the notion that women tend to prioritize family responsibilities while men prioritize their work,^22-24^ our results indicate similar effects between the sexes. It has been argued that work-related stressors are more linked to WFC, while family stressors are more associated with FWC.^3^ FWC - which occurs when it is difficult to meet work expectations due to family obligations^1,2^ - is a less-explored WFC dimension in the international literature; we could find no study that associated this dimension with SRH trajectories. In any case, the results may vary according to the cultural and social context, family and work experience, and the ways in which men and women deal with challenges. Some factors, such as the significance of domestic and professional work roles, family composition, the extent to which workers share family responsibilities, and the extent to which they consider work and family roles independent or interdependent, may explain these findings, although they were not evaluated in the present study. Future studies could explore these issues in greater depth.

The association between lack of time for self-care and leisure and worse SRH trajectories should be considered in light of gender issues and the modifying role of education. Significant associations between these variables were observed in both men and women, although among women the associations were limited to those with a high education level. A perceived lack of time seems to be part of contemporary life, in which workers must be versatile and multifunctional.^25^

While attempting to fulfill the demands of work and family, adults can relegate their health to the background, even unconsciously. Prioritizing work and family activities can reduce sleep time and leisure activities, increasing psychological stress and, consequently, affecting health.^26^ A perceived lack of time may lead to postponing or missing doctor visits, which could lead to later disease diagnosis.^27^

This study showed that women with a high education level who reported a lack of time for self-care and leisure were more likely to have a worse SRH trajectory. The occupations of people with a lower education level are generally more physical and involve well-defined work shifts and less autonomy. In general, these workers clock in, work a set number of work hours, and “disconnect” from work when they return home. In contrast, workers with a high education level, who are more likely to have higher incomes, may have greater difficulty disconnecting from work because they tend to perform jobs with greater qualitative demands, which are associated with greater stress and longer hours without well-defined schedules. This can contribute to a greater risk of WFC.^28^ The proliferation of communication technologies has broken down many barriers between work and personal life in highly demanding occupations.^29^

The fact that education level affected the perceived lack of time for self-care and leisure among women was, in addition to the above mentioned factors, due to combining these factors with domestic activities. Although the professional labor market is increasingly occupied by women, men are still not equally involved in domestic activities.^22-24^

Thus, while trying to fulfill the demands of work and home, women sacrifice time for self-care, which has health consequences. Although the literature shows that low income, education, and job positions are significantly associated with lower SRH and greater WFC,^30^ cross-sectional analyses based on ELSA-Brasil data have already shown associations between WFC and poor SRH among women with a high education level.^12^

The effects of a high education level could be explained by the worker profile in this cohort, which consisted mainly of university and research institution employees who perform highly controlled and demanding work. In addition, these employees work long hours and have flexible schedules, which makes it difficult to establish clear boundaries between work and personal life.^12^

One strength of our study was that the associations are based on longitudinal data, including 11 measures of SRH over time. In addition to using a bidirectional concept of WFC, another important point was the use of an indicator that measures the degree to which work and family can interfere with perceived time for self-care and leisure. In addition, the effect-modifying role of education in longitudinal analysis deserves special attention, since some cross-sectional studies have found significant associations only among women with low education, while others have found them among women with high education.

Although data on WFC and other covariates were only used at baseline, it is assumed that no major changes occurred over time for these variables, since ELSA-Brasil includes a population of public servants with high occupational stability. It is important to note that because our study population consists of public servants, the findings may not be representative of the general population or other groups of workers. However, there was sufficient socioeconomic variability to capture differences in sex and education.

## CONCLUSIONS

In summary, this study contributes to the literature regarding the association between different domains of WFC and SRH trajectories over time, in addition to sex differences and the relevant role of education for women. The results showed an association between WFC and the worse SRH trajectories, with more consistent effects among women than among men. Education proved to be an important effect modifier, since a lack of time for self-care and leisure and worse SRH trajectories were associated only among women with high education.

The results of this study underscore the need for a more equitable division of housework between men and women, which could reduce WFC and the stress it entails, in addition to positively influencing worker priorities regarding the use of time for self-care, thus improving the health and well-being of all. It is also necessary to develop public policies that guarantee the well-being of workers, reduce gender inequality, and establish more defined limits between personal and professional spheres, especially for highly educated female workers.
